# Special Issue “Mitochondrial Dysfunction: A Common Trigger in Neurodegenerative and Metabolic Non-Communicable Diseases”

**DOI:** 10.3390/ijms25074004

**Published:** 2024-04-03

**Authors:** Paola Fortini, Barbara Pascucci

**Affiliations:** 1 Department of Environment and Health, Istituto Superiore di Sanità, Viale Regina Elena 299, 00161 Rome, Italy; 2 Institute of Crystallography, Consiglio Nazionale delle Ricerche, Strada Provinciale 35d, n.9, Montelibretti, 00010 Rome, Italy

Non-communicable diseases (NCDs) are non-infectious and non-transmissible chronic disorders. They include cancer, neurodegenerative, autoimmune and cardiovascular diseases, as well as metabolic dysfunctions, such as diabetes and obesity. NCDs are characterized by low-grade inflammation and oxidative stress and are frequently associated with a marked mitochondrial dysfunction [1]. Mitochondrial functionality is the result of a fine-tuned balance between biogenesis, dynamics, and mitophagy [2,3,4]. The impairment of this quality control leads to the engulfment of cells in tissues and organs with dysfunctional organelles, causing their release into the cytosol and bloodstream of several mitochondrial molecules, collectively known as damage-associated molecular patterns (DAMPs) [5]. Mitochondrial DAMPs include proteins (TFAM and N-formylpeptides), lipids (cardiolipin), metabolites (succinate and ATP), and mitochondrial DNA [6]. They can activate the innate immune response, thereby contributing to the development of chronic inflammatory disorders [7].

This Special Issue, entitled “Mitochondrial Dysfunction: A Common Trigger in Neurodegenerative and Metabolic Non-Communicable Diseases” of the *International Journal of Molecular Sciences*, includes a total of seven contributions, composed of four research articles and three reviews. New information on the role of mitochondria and oxidative stress in both neurodegenerative and metabolic diseases, as well as in brain tumors, has been presented. The contribution of mitochondrial dysfunction has also been investigated in acute anoxia and preeclampsia.

Spermidine is an ubiquitary polyamine with well-known geroprotective properties. It can extend the health span and lifespan of different organisms, from fungi to mammals, and delay the onset of cardiovascular diseases and neurodegenerative disorders [8]. At the molecular level, spermidine seems to be a natural autophagy inducer [9]. Fairley and coworkers (contribution 1) investigated the specific role of spermidine on mitochondrial dysfunction induced by the deposition of abnormal hyper-phosphorylated tau protein, a microtubule protein. The hyper-phosphorylation of tau protein causes its dissociation from microtubules with the consequent formation of insoluble aggregates and neurofibrillary tangles, the hallmark of tauopathies, a class of human neurodegenerative diseases that includes Alzheimer’s disease (AD) and Parkinson’s disease. Using the human neuroblastoma SH-SY5Y cell line, a cellular model of tauopathies, the authors determined the effects of spermidine on the bioenergetics and cell metabolic activity versus the control counterpart, SH-SY5Y cells carrying the empty vector. They showed that spermidine improved mitochondrial oxidative phosphorylation, mitochondrial membrane potential, and ATP production in tau-expressing cells. Moreover, spermidine treatment decreased the level of free radicals, increased autophagy, and restored tau-induced impairments in mitophagy, inducing the gene expression of players involved in these mechanisms, *lc3*, *p62*, and *parkin*. The authors suggest that spermidine has potential therapeutic properties to counteract tau-related mitochondrial dysfunction.

Jastroch’s research group (contribution 2) investigated the role of the mitochondrial dynamics in glucose intake-dependent insulin secretion by pancreatic cells. It was already known that altered mitochondrial morphology, due to the different levels of expression of the genes involved in fusion and fission machinery, is associated with glucose-stimulated insulin secretion in β pancreatic cells. They investigated the crosstalk between mitochondrial dynamics and insulin secretion efficiency in pancreatic cells, studying the role of Drp1, the major regulator of mitochondrial fission. Unexpectedly, the overexpression of Drp1 in wild-type MIN6 pancreatic cells decreases insulin content and failed to rescue insulin secretion to wild-type levels in Drp1 knockdown cells. The authors suggest that the reduced insulin secretion observed in MIN6 cells is due to the Drp 1-dependent impairment of insulin biosynthesis by the activation of the PKA/eIF2α/Fgf21 pathway. The PKA/eIF2α/Fgf21 pathway is responsible for endoplasmic reticulum stress and consequently reduced protein translation. Therefore, the authors claimed that attention should be paid using the overexpression of Drp1 as a therapeutic tool, considering the side effects on insulin biosynthesis.

To understand whether mitochondrial morphological alterations and dysfunction are early or late events in brain malfunctions, Morozov and coworkers (contribution 3) investigated the structure and reorganization of both mitochondria and Golgi apparatus (GA) in embryonic mouse brain during acute anoxia. Analysis using electron microscopic three-dimensional reconstruction showed that the GA was the most vulnerable organelle, showing a clear deformation already after 1 h of anoxia, whereas the mitochondria maintained the normal ultrastructure. Only after 3 h of anoxia did the mitochondria assume an unconventional structure. The authors suggest that anoxia-induced GA disorganization may be responsible for the mitochondrial dysfunction observed at longer treatment times. Currently, this is the first study which suggests a potential link between GA phenotype and mitochondrial functionality.

Furthermore, the potential contribution of mitochondrial genome changes in brain tumor occurrence has been investigated by Kozakiewicz and coworkers (contribution 4). Genes encoding for respiratory chain components were analyzed by next-generation sequencing for DNA polymorphisms and mutations in blood and tumor biopsies of a cohort of 30 Caucasian patients, diagnosed with WHO grade II, III, or IV glioma. In particular, the consequences of missense mutations, able to alter the structure and biochemical properties of encoded proteins, were investigated by in silico studies. Only mitochondrial cytochrome b alterations are frequently found and seem to play a role in brain glioblastoma formation.

Xu and colleagues (contribution 5) thoroughly reviewed recent studies highlighting the crucial role of mitochondrial dysfunction in adipose tissue macrophages (ATMs) in obesity, a phenotype commonly associated with type 2 diabetes and insulin resistance. The hypertrophic expansion of adipose tissue creates local hypoxia and the release of fatty acids, leading to the accumulation of dysfunctional mitochondria in ATM. The impairment of mitochondrial quality control leads to the release of mitochondrial DAMPs responsible for the activation of the NLRP3 inflammasome with the subsequent release of proinflammatory cytokines. Interestingly, therapeutic strategies aimed at restoring homeostasis and reprogramming metabolism in ATM for the treatment of diabetes and insulin resistance have also been discussed.

Finally, Veselov et al. and Jahan and colleagues presented a growing body of evidence on the key role of mitochondrial dysfunction as a common trigger in AD and type 2 diabetes mellitus, as well as in the context of preeclampsia, highlighting how the loss of mitochondrial homeostasis can contribute to the onset and chronicity of apparently very distant NCD (contributions 6 and 7).

In conclusion, the contributions included in this Special Issue explore several aspects and different pathological conditions in which mitochondrial dysfunction seems to play a role and shed more light on many unresolved questions in this field. However, much remains to be done, mainly in terms of attempting to translate research findings into therapeutic approaches in neurodegenerative and metabolic NCDs.

Nevertheless, no studies in this Special Issue addressed microbiota–mitochondrial crosstalk. In recent years, several studies have strongly suggested a pivotal role of the microbial communities in numerous NCDs [10], and bidirectional crosstalk between gut microbiota and mitochondria has recently been discovered [11]. Gut microbiota by-products can modulate the gene expression levels of players with a role in the mitochondrial biogenesis and metabolic pathways of host cells, such as PGC-1α AMPk and SIRT1 [12]. On the other hand, the increase in mitochondrial ROS, due to oxidative phosphorylation impairment, leads to redox imbalance and gut barrier integrity loss [13]. Future research, focusing on the fine characterization of the molecular mechanisms involved in the interplay between mitochondria and microbiota in the host cells, could pave the way to new therapeutic approaches and should therefore be encouraged.
